# Games to Improve the Clinical Skills of Nursing Students: Systematic Review of Current Evidence

**DOI:** 10.2196/70737

**Published:** 2025-08-11

**Authors:** Soleiman Ahmady, Hanieh Zehtab Hashemi, Arghavan Afra, Niloofar Attarian, Amir Tabari, Fariba Shabani, Ayoob Molla, Esmaeil Mehraeen

**Affiliations:** 1 Department of Medical Education, School of Medical Education and Learning Technologies, Shahid Beheshti University of Medical Sciences, Tehran, Iran; 2 Department of Health Informatics, Smart University of Medical Sciences, Tehran, Iran; 3 Artificial Intelligence in Medical Sciences Research Center, Smart University of Medical Sciences, Tehran, Iran; 4 Department of Nursing, School of Nursing, Abadan University of Medical Sciences, Abadan, Iran; 5 School of Medicine, Azad University of Medical Sciences, Mashhad, Iran; 6 Department of Health Information Management, School of Allied Medical Sciences, Shahid Beheshti University of Medical Sciences, Tehran, Iran; 7 School of Medicine, Bushehr University of Medical Sciences, Bandar Būshehr, Iran; 8 Department of Medical Education, Smart University of Medical Sciences, No.3 1st Alley, Sarafraz St, Shaheed Beheshti St, Tehran, Iran, 98 218850405, 98 2188501409

**Keywords:** education, nurse education, educational games, game-based learning, simulation, clinical skills, nursing

## Abstract

**Background:**

As medical education evolves, incorporating innovative teaching methods is crucial for developing nursing students’ critical thinking and problem-solving skills. Game-based learning (GBL) has gained popularity, engaging students through immersive experiences and allowing personalized learning.

**Objective:**

This systematic review aimed to investigate the impact of educational games on outcomes of clinical nursing skills.

**Methods:**

In this study, the authors systematically searched the 4 public databases (PubMed, Embase, Scopus, and Web of Science) to investigate the role of educational games in improving the clinical skills of nursing students. This paper is based on the PRISMA (Preferred Reporting Items for Systematic Reviews and Meta-Analyses) 2020 guidelines. We also checked the bias risk of selected studies by the Newcastle-Ottawa Scale (NOS) bias assessment tool.

**Results:**

In this study, 801 articles were initially retrieved using a specified search strategy, with 38 remaining after applying inclusion and exclusion criteria. The final included studies published between 2017 and 2023 spanned various countries and focused on diverse learning objectives. A broad range of learning objectives, such as developing diagnostic reasoning, enhancing knowledge and cognitive skills, and improving training methods, can be supported by a game-based platform. We also showed that while many games used web-based platforms, few were conducted in person, and some were developed in app formats for smartphones.

**Conclusions:**

GBL is transforming nursing education by enhancing student engagement and clinical skills through immersive experiences. Despite its advantages, GBL faces challenges such as development costs and the effect of expertise reversal. Developing standardized assessment tools will help unify definitions and improve the comparability of research findings, ultimately enhancing the evidence base for GBL’s effectiveness.

## Introduction

As medical science and medicine advance daily and continuously influence medical education, health care, and nursing must constantly seek innovative teaching and learning methods [1-3]. Nurses are the frontline of medical care and constitute the largest segment of the global health workforce. Considering this, it is more than crucial for nursing students to receive a thorough education during their training, which can be provided through advanced evidence-based educational programs that keep pace with current changes in the field [4].

Currently, medical and nursing students are required to acquire advanced skills during their educational courses, including high levels of skills in critical thinking, problem-solving, and analysis, to pave the way toward developing higher-order thinking skills [1]. Nursing education encounters several challenges, including limited training time, which restricts the ability to provide effective practical learning experiences. These obstacles underscore the necessity for innovative strategies that can complement traditional educational methods and improve the overall quality of nursing education [5]. With the emerging role of technologies in this century, it is essential to incorporate active learning methods that go beyond traditional reading, listening, or watching, enabling students to explore and integrate knowledge actively to develop these levels of skills [1].

E-learning, web-based learning, computer-based simulators, mobile devices, virtual reality modalities, and games are a few of the various information technologies and educational methodologies that have been incorporated into nursing education due to technological advancements that we are witnessing in the current era, augmenting the traditional educational curricula [4]. Among the diverse modalities of technology-enhanced learning, games serve as exemplary tools for facilitating collaborative activities, review, and evaluation within the classroom setting. They introduce an element of enjoyment and maintain a clear educational objective, resulting in positive learning outcomes. Furthermore, microlearning (the process of acquiring knowledge through small chunks of information that can be delivered via videos, articles, ebooks, audio clips, etc) strategies contribute to enhancing the effectiveness of educational experiences [6].

The application of gamification within the education of health care professionals is experiencing rapid growth [7]. Gamification is a strategic approach to enhance engagement by integrating game elements into educational settings. This methodology aims to develop specific skills, establish objectives that imbue learning with meaning, actively involve students, optimize the learning process, facilitate behavioral change, and promote social interaction [8].

In nursing education, there has been a notable increase in the adoption of innovative teaching methods to adapt to the aforementioned issues. One such approach that is becoming increasingly and significantly popular is game-based learning (GBL) [1]. GBL incorporates games or game-like elements, concepts, mechanisms, or designs into education. This approach integrates educational games into classroom instruction and self-directed learning, offering students an immersive learning experience that enhances their acquisition of knowledge and skills [9]. This method consists of enabling students to learn through personal experiences via gaming perspectives and platforms and allows them to manage their learning according to their progress and capabilities [1].

GBL research often references three key terms: “serious game,” “educational game,” and “digital educational game.” These terms overlap, but differ in specific ways. A serious game refers to games designed with an educational purpose, rather than for entertainment. Educational games are games specifically designed for education, including both physical and digital formats. Narrowly defined, they are electronic games developed for learning, while broadly defined, they encompass traditional games, educational software, and toys that combine fun with learning objectives. In addition, digital educational games refer to educational games that require digital platforms and technology. They promote understanding of educational content and come in various types, such as adventure, role-playing, business, and logic games. These games can support both single-player and multiplayer formats. This concept can integrate fun with learning, enhancing the educational experience by leveraging technology to engage learners and improve their mastery of the subject matter [9].

These approaches support learning by integrating activities like feedback, testing, and spaced repetition with active participation, autonomy, and positive experiences for students, promoting a more effective educational environment [10]. According to the literature available surrounding this matter, students who have experienced GBL are more likely to achieve significantly better cognitive learning outcomes. GBL creates an atmosphere of high enthusiasm, active participation, and great enjoyment, and the fusion of these is more likely linked to improving learning results [11].

By fully engaging in an activity, one can achieve a state of intense focus, which can aid the learning process and lead to an optimal experience of learning. This state is referred to as “flow.” GBL encompasses physical games like board games and digital formats, including virtual reality. These educational models engage students with challenges that promote exploratory behavior, allowing them to actively solve problems and reflect on their actions, drawing on the concept of “flow” [1]. The affordances identified in GBL are consistent with the principles of flow theory. It is anticipated that as players engage with the game and refine their skills, the level of difficulty will progressively increase. Consequently, flow theory is used in game studies to foster concentration among students during lectures, based on these principles [12].

Although nurse educators have been slow to embrace this concept, research shows that game-based learning frequently outperforms traditional teaching methods [1,7]. In addition, the COVID-19 pandemic emerged as a cause to force higher education institutions to rapidly shift to virtual teaching and learning, which necessitated maintaining student engagement while ensuring that the content and methods stayed relevant to their future careers [7].

A recent systematic review supports these points and emphasizes the necessity for more research on the use of specific games among nursing students [1]. Identifying and analyzing the increasing number of published studies in this area is important to develop a comprehensive evidence base. This systematic review aims to identify studies on educational games in nursing education that enhance students’ skills, summarize their impacts on learning outcomes, and explore the concept of game-based learning in nursing.

## Methods

### Overview

In this study, the authors systematically searched the 4 public databases to investigate the role of educational games in improving the clinical skills of nursing students. This paper is based on the PRISMA (Preferred Reporting Items for Systematic Reviews and Meta-Analyses) 2020 guidelines (the PRISMA checklist is provided in Checklist 1). We also checked the bias risk of selected studies by the Newcastle-Ottawa Scale (NOS) bias assessment tool.

### Data Sources

We searched the defined keywords in the databases of PubMed, Embase, Scopus, and Web of Science. The search was conducted on July 14, 2024, and all English-language studies were included in the screening. The PubMed search query and its combinations are as follows: (“games, experimental”[MeSH Terms] OR “gamification”[MeSH Terms] OR “game”[Title/Abstract] OR “gamification”[Title/Abstract] OR “educational game”[Title/Abstract] OR “serious game”[Title/Abstract]) AND (“Nurses”[MeSH Terms] OR “Nursing”[MeSH Terms] OR “Nurse Practitioners”[MeSH Terms] OR Nurses[Ti] OR Nursing[Ti] OR “Nurse Practitioners”[Title/Abstract]) AND (“Education”[MeSH Terms] OR “Education”[Title/Abstract] OR “Learning”[Title/Abstract] OR “Nursing education”[Title/Abstract] OR “Clinical skills”[Title/Abstract] OR “Virtual reality”[Title/Abstract] OR “Virtual learning”[Title/Abstract] OR “Virtual education”[Title/Abstract] OR “Virtualization”[Title/Abstract]). Similar structured queries were applied in Embase, Scopus, and Web of Science, adjusted for each database’s indexing system (Multimedia Appendix 1).

(“games, experimental”[MeSH Terms] OR “gamification”[MeSH Terms] OR “game”[Title/Abstract] OR “gamification”[Title/Abstract] OR “educational game”[Title/Abstract] OR “serious game”[Title/Abstract]) AND (“Nurses”[MeSH Terms] OR “Nursing”[MeSH Terms] OR “Nurse Practitioners”[MeSH Terms] OR Nurses[Ti] OR Nursing[Ti] OR “Nurse Practitioners”[Title/Abstract]) AND (“Education”[MeSH Terms] OR “Education”[Title/Abstract] OR “Learning”[Title/Abstract] OR “Nursing education”[Title/Abstract] OR “Clinical skills”[Title/Abstract] OR “Virtual reality”[Title/Abstract] OR “Virtual learning”[Title/Abstract] OR “Virtual education”[Title/Abstract] OR “Virtualization”[Title/Abstract])).

### Study Selection

Articles related to the purpose of our study were screened and selected in 2 steps. In the first stage, the titles and abstracts of the studies were evaluated, and the relevant articles were selected for the second and deeper stage according to their titles and abstracts. In the second step, the authors reviewed the full texts of these articles. Publications that met the inclusion criteria were selected for data extraction (Article selection criteria Textbox 1).

Textbox 1. **Inclusion criteria:**

Papers reporting educational games in nursing, published in the English language, and being original.

**Exclusion criteria:**

Articles without available full texts, lacking published data, duplicated articles, case series, case reports, conference abstracts, and letters to the editors.

### Data Extraction

After selecting eligible articles, data extraction began. A total of 4 researchers diligently reviewed the full texts of the selected studies and pulled the required data together. Data related to the objectives of this research were extracted from selected studies. Data about the first author, country, year of publication, the aim of the study, participants, learning objectives, type and name of the game, platform, game features, and main findings of selected studies were extracted in Tables1  2.

**Table 1.** Specifications of included articles.

ID	The first author (Reference)	Country (Year)	The aim of the study	Participants (number)
1	Tinoco JD [13]	Brazil (2023)	Develop and assess a board game’s effectiveness for teaching diagnostic reasoning among nursing students.	19 experts and 11 undergraduate nursing students
2	Vazquez-Calatayud M [14]	Spain (2023)	To enhance postgraduate nursing students’ decision-making skills using a game-based learning intervention.	66 postgraduate nursing students.
3	Morgan DJ [15]	United States (2023)	Conducting an online game-based learning (GBL) that uses natural frequencies and feedback to teach diagnostic reasoning.	90 students
4	Morrell B [16]	United States (2020)	Conducting a cardiovascular-themed educational escape room for undergraduate nursing students, outlining its goals, design, and materials for easy adaptation to other curricula.	31 to 68 students
5	Morrell B [17]	United States (2020)	A study was conducted to explore baccalaureate nursing students’ perceptions of a cardiovascular-themed educational escape room.	57 students
6	Kubin L [18]	United States (2020)	Evaluation of the effectiveness of escape room activities as educational tools in nursing education.	129 students
7	Blanie A [19]	France (2018)	This trial compared the effectiveness of gaming simulation (SG) versus traditional teaching (TT) for improving clinical reasoning (CR).	146 students
8	Gómez-Urquiza JL [20]	Spain (2020)	Aimed to explore nursing students’ opinions about an escape room-based game as an evaluation game	105 students
9	Baek G [21]	Korea (2023)	Development of a program regarding cardiopulmonary resuscitation training via a web-based serious game for nursing students.	A total of 44 participants
10	Farsi Z [22]	Iran (2018‐2019)	This study compared traditional mannequin-based CPR^a^ training with innovative smartphone-based serious game training for nursing students.	56 nursing students
11	Fijacko N [23]	Slovenia (2023)	To assess the effectiveness of the MOBICPR game in improving nursing students’ theoretical knowledge and practical skills in adult Basic Life Support (BLS).	43 nursing students participated in the study.
12	Guti´errez-Puertas L [24]	Spain (2019)	Design and develop an app that aids nursing students in acquiring knowledge of Basic and Advanced Life Support Techniques.	184 students
13	Elzeky M [25]	Egypt (2020‐2021)	Evaluation of the impact of gamified flipped classrooms on nursing students’ competency and learning motivation	128 nursing students
14	Bayram ŞB [26]	Turkey (2021)	To improve nursing students’ knowledge and understanding of tracheostomy care.	125 nursing students completed both the pretest and post-test
15	Breitkreuz K R [27]	United States (2019)	To assess the usability of the VR^b^ Sterile Urinary Catheter Insertion Game (VRSUCIG) and nursing student reactions.	300 pre-licensure nursing students from nine schools.
16	Chan K [28]	China (2021‐2022)	The study evaluated VR-Hospital’s impact on nursing students’ skills, satisfaction, self-confidence, and overall experience.	202 students
17	Hwang JG [29]	Taiwan (2020)	A contextual game-based flipped learning approach (GBFL)	56 students
18	Jung SY [30]	Korea (2022)	Comparing the feasibility and learning outcomes of a novel pressure ulcer management VR simulation program’s feasibility with video lectures.	35 novice nurses
19	Koivisto JM [31]	Finland (2018‐2019)	The objective was to assess the link between game metrics in a simulation and the surgical nursing knowledge of students.	280 students
20	Koivisto JM [32]	Finland (2018‐2019)	This study aimed to assess how a simulation game impacts nursing students’ surgical knowledge.	385 students
21	Lau ST [33]	Singapore (2021)	This study examines immersive virtual reality (IVR) clinical procedures on mid-career switch students’ knowledge, perceptions, and experiences.	34 students
22	Kulakc N [34]	Turkey (2020)	An RCT^c^ was conducted to examine the effects of a serious game-based web application on stoma care education for nursing students.	98 students
23	Nasirzade A [35]	Iran (2022)	This study compared the impact of feedback lectures and the BAM^d^ Game on nursing students’ knowledge and skills in burn patient assessment	42 students
24	Karci HD [36]	Turkey (2023)	The study examines the role-play gamification’s impact on nursing students’ skills.	10 students
25	Wang Z [37]	China (2021)	Aimed to assess the effectiveness of a mobile game app in enhancing ECMO pipeline pre-flushing skills among critical care specialist nurses.	86 intensive critical care specialist nurses
26	Chang CY [38]	Taiwan (2019)	The aim was to develop an RPG (role-playing game) to enhance nursing students’ performance in Electrocardiogram training	72 4th-year nursing students
27	de Beer E.H.M [39]	Netherlands (2018‐2019)	The study explored how nursing students perceive collaborative problem-solving (CPS) skills development through assignments in the hybrid serious game Carion.	181second-year bachelor nursing students (19males and 162females).
28	Wong JYH [40]	China (2022)	To develop and evaluate a serious game, Virtual Emergency Room (ER), aimed at enhancing teamwork attitudes and clinical competency among medical and nursing students during emergency care scenarios.	62 final-year medical and nursing students.
29	Ropero-Padilla C [41]	Spain (2020‐2021)	The study aimed to explore nursing students’ experiences and perceptions of using game elements in two nursing courses through a blended-learning approach.	149 students
30	Calik A [42]	Turkey (2021)	To evaluate the effectiveness of a serious game (SG) in improving senior nursing students’ knowledge and understanding of COVID-19, including personal protective equipment (PPE) use, quarantine/isolation periods, and symptoms.	62 participants in the final analysis.
31	Wu SH [43]	Taiwan (2017‐2019)	To evaluate the effectiveness of a VR game-based training system for preventing needle stick or sharp injuries (NSI) among new nursing and medical interns.	109 participants (59 nursing interns, 50 medical interns).
32	Al-Mugheed K [44]	Cyprus (2019)	Aimed to assess the impact of online learning and game-based virtual reality apps on standard precautions.	126 students
33	Mitchell G [45]	United Kingdom (2018‐2019)	to assess how a ’serious game’ about influenza impacts nursing students’ attitudes, knowledge, and adoption of the influenza vaccine.	430 students
34	Ma Z [46]	United States (2021)	The study aimed to assess how feasible and effective a computer role-playing game (CRPG) is for enhancing nursing students’ empathy, emphasizing immersion and perspective.	69 students
35	Rodriguez-Ferrer JM [47]	Spain (2020‐2021)	This research evaluated escapes rooms as a strategy to reduce stigma toward serious mental disorders.	197 students
36	Chen D [48]	China (2023)	To evaluate the impact of an Escape room (ER) game on nursing students’ learning attitudes and game flow experience in a Gerontological Nursing course.	83 nursing students (41 in the test group, 43 in the control group).
37	Idrissi W [49]	Morocco (2022)	This study aims to explore how serious games affect nursing students’ learning, engagement, and motivation.	58 polyvalent nursing students
38	Labrague L [50]	United States (2023)	Teaching delegation skills, a critical competency in nursing, through interactive scenarios.	N/A

a CPR: cardiopulmonary resuscitation.

b VR: virtual reality.

c RCT: randomized controlled trial.

d BAM: burn assessment mission.

**Table 2.** Overview of the included studies that address games for nursing education.

ID	Learning objectives	Type/Name of the game	Platform	Game features	Main findings
1	Develop diagnostic reasoning in nursing	Enfermeiro Diagnosticador	N/A^a^	search and organization of clues capable of evidencing the nursing diagnosis presented by patients	“Enfermeiro Diagnosticador” is effective in supporting the teaching of diagnostic reasoning in nursing
2	Improve decision-making in clinical scenarios, particularly in conflict situations and high-risk protocols.	A combined case-based learning and escape room game	Conducted in a real-world setting with physical escape rooms	Case-based learning scenarios, escape room challenges, puzzles, and ethical dilemmas	High student engagement, improved decision-making skills, and positive feedback on learning effectiveness and enjoyment
3	Teaching diagnostic reasoning	Diagnostic reasoning game with natural frequencies feedback	Online via a web browser	Estimates diagnosis probability, provides immediate feedback, and includes a tutorial	A single GBL^b^ session almost doubled diagnostic accuracy scores in medical trainees and clinicians, with lasting effects for three months
4	Enhance understanding of cardiovascular conditions and clinical reasoning through interactive problem-solving	Cardiogenic Shock Educational Escape Room	Traditional classroom setup	Nine puzzles in a mock hospital; time-limited, with feedback	This game enables students to pursue a shared objective by collaborating, providing feedback, and seeking assistance
5	Enhance understanding of cardiovascular conditions	Live-action cardiovascular critical care escape room	In person	Teams of 4‐5 solve cardiovascular puzzles in 60 min, unlocking a final box to stop a countdown	The escape room-based learning method may be one way to enhance students’ professional practice skills
6	Enhancing clinical reasoning, problem-solving, and collaboration among nursing students	Escape Room	N/A	Solving puzzles, riddles, and NCLEX-style^c^ questions	Escape rooms can be utilized as an effective augmentation to traditional learning methods
7	Improving clinical reasoning in nursing students	LabForGames Warning	Played on computers with a 3-D interactive environment	Students practice clinical scenarios, detect deterioration, and communicate using SBAR^d^	This study found no significant CR^e^ difference between SG^f^ and TT^g^, but SG increased satisfaction and motivation
8	N/A	Nursing Escape Room	In-person escape room at the University of Granada	Find a fake nursing document in 30 min using clues and a classroom key	The escape room learning game provided a positive experience for students, noted for being enjoyable, engaging, and motivating
9	Cardiopulmonary resuscitation training in nursing students	Advanced Cardiac Pulmonary Resuscitation (ACPR)	Web-based platform hosted on Amazon Web Services (AWS)	A realistic cardiac resuscitation game with sequential learning, interactive 3D environment, and personalized feedback	The program effectively enhanced nursing students’ cardiopulmonary knowledge, confidence, problem-solving, and learning transfer
10	Improving CPR training and skills	Serious game for CPR training	N/A Using smartphones	Self-learning, step-by-step instructions, self-assessment, feedback on tasks, retry options, and communication with researchers via group chat	Both simulation and serious game training improved CPR^h^ skills, suggesting a multimodal educational approach could be beneficial
11	To enhance the participants’ theoretical knowledge and practical skills in adult BLS, particularly their ability to perform cardiopulmonary resuscitation (CPR^h^) and use an automated external defibrillator (AED)	MOBICPR	The game was played on a Samsung Galaxy A13 smartphone	The game simulates BLS for out-of-hospital cardiac arrest (OHCA) using gestures, scores, and 2021 European Resuscitation Council (ERC) guidelines to teach CPR^h^	MOBICPR significantly improved BLS knowledge and skills, suggesting serious games enhance nursing students’ BLS training
12	Enhancing students’ knowledge of Basic and Advanced Life Support Techniques	Guess It (SVUAL)	Mobile app	Phases of keyword guessing, retention testing, and knowledge reinforcement based on life support techniques	The app has demonstrated high content quality and user-friendliness, enhancing nursing students’ knowledge and information retention
13	Enhance nursing students’ competency and learning motivation	Gamified Flipped Classroom (FC) for Nursing Fundamentals	Moodle platform with additional gamification features	Includes quizzes with varying difficulty levels, badges, leaderboards, ranks, levels/unlocks, and points	Gamified flipped classrooms boost nursing students’ motivation, preparation, skills, knowledge, and confidence in lab practice
14	Learning tracheostomy care and remember their prior knowledge	Tracheostomy care knowledge test (TCKT)	The game was available on a dedicated website [51] and could be played on a computer	The game is a 10 min interactive tool consisting of six stages. The player must complete each stage correctly to progress to the next	The game effectively improved tracheostomy care knowledge, especially for first-year students, enhancing learning enjoyment and realism
15	To practice sterile urinary catheter insertion skills using VR^i^	VR Sterile Urinary Catheter Insertion Game (VR-SUCIG)	N/A	Virtual feedback on technique, scoring, cue cards, and visual cues for contamination	The game had medium usability; students found it engaging and effective for practice
16	Enhancing nursing students’ skills, satisfaction, self-confidence, and overall experience	Virtual Reality Hospital (VR-Hosp)	HTC Vive Cosmos	Single-user VR game with 3D ward, scenarios, speech recognition, randomized tasks, and NTS^j^ development	The positive outcomes provide a foundation for developing IVR^k^ activities in nursing education
17	Exploring the situation of intravenous injection flipped learning	Intravenous Injection Game	N/A	Game-based learning, decision-making scenarios, skills practice, and group discussions	Students using the new method excelled in intravenous injection comprehension, motivation, and critical thinking compared to conventional methods
18	Improving pressure ulcer management training	PU-VRSim^l^	Unity 3D	The game simulates pressure ulcer care, enhancing critical thinking, self-efficacy, and clinical judgment using VR	PU-VRSim enhances novice nurses’ PU^m^ management skills
19	Improving surgical nursing knowledge	Simulation game with surgical nursing scenarios	Desktop virtual simulation	Simulation game with realistic nursing scenarios, learning-enhancing elements, and flexible scoring system for educational research	Higher game scores correlated with better surgical nursing knowledge; time spent playing had no impact
20	Improving surgical nursing knowledge	3D simulation game developed with Unity	N/A	Includes five surgical patient scenarios, realistic graphics, animations, and interactive elements for interviewing, assessing, and implementing nursing interventions	This study demonstrates that a simulation game effectively enhances nursing students’ knowledge in surgical nursing
21	Enhancing intravenous therapy and insulin therapy skills	IVR clinical procedures simulation	The participants used Meta Quest 2 VR headsets	IVR simulation for intravenous therapy and insulin with 3D avatars, practice/assessment modes, and virtual patients	IVR simulation can enhance clinical procedure knowledge in mid-career students
22	Enhancing stoma care and colostomy irrigation knowledge	N/A	Web-based application	The game allowed students to practice stoma care and colostomy irrigation skills in a simulated environment, reinforcing the theoretical knowledge they had gained	Students’ knowledge and skill scores improved significantly as they spent more time with the serious game
23	Learning of burn patient assessment	BAM^n^ Game	Web-based game accessible via computers or mobile devices	Interactive game: burn assessment, real images, videos, feedback, competitive scoring	Nursing students using the BAM game excelled in knowledge and skill development
24	Enhancing skills related to internal medicine	Role-Play-Based Gamification	N/A	Role-playing various medical scenarios, developing nursing skills, enhancing communication, incorporating scoring systems and symbolic rewards, and embedding fun and humor to facilitate learning	Gamification enhanced students’ motivation and retention in learning nursing care and communication skills
25	improving ECMO pipeline preflushing skills	N/A	Mobile phones	Interactive stages simulating ECMO pipeline preflushing include 61 steps with real-time feedback and scoring	This study indicates that a game-based mobile app could be more effective than traditional Chinese lecture-practice methods for teaching ECMO pipeline preflushing to critical care specialist nurses
26	Enhance ECG^o^ training in nursing	ECG Clinical Context Role-Playing Game	RPG^p^ Maker MV by Enterbrain Incorporated	This ECG game uses RPG Maker MV to enhance learning with challenges, fights, and storylines	Students using this game outperformed those in traditional instruction in learning, attitude, and critical thinking
27	The primary learning objective was to improve students’ collaborative problem-solving skills, encompassing both social and cognitive skills	The game is called Carion	The game uses multiple platforms: Wijklink (an online collaboration platform), Blackboard (the university’s online learning environment), in-school learning environments, and real-life district assignments	One semester (20 wk, 840 study hours)	Students used each other’s strengths to enhance teamwork and problem-solving through innovative serious games
28	(1) Competency in history taking and physical assessment skills; (2) diagnostic skills, alertness, and treatment skills; (3) clinical procedural skills and prioritization during emergency situations. (4) professionalism, responsibility, and a caring attitude	Virtual Emergency room (ER)	Web-based application created with Tumult Hype, played online on laptop	Interactive emergency care game with roles, scoring, and feedback to enhance learning and clinical skills	Virtual ER improved teamwork attitudes; activists and pragmatists benefited most; students rated it positively
29	N/A	N/A	Google Meet and Google Classroom for online sessions	Game elements in blended learning were assessed via focus groups, with online gamified activities and rewards	This study presents new findings on using game elements in blended learning, demonstrating their effectiveness in teaching key clinical and teamwork skills like creativity, communication, and responsibility
30	The learning objectives included understanding virus incubation time, recognizing COVID-19 symptoms, proper donning and removing PPE^q^, and knowledge about quarantine and isolation periods	N/A	N/A	The game featured login, avatars, progress tracking, PPE, symptoms, and quarantine management, developed on Articulate	Students’ knowledge improved significantly on isolation times and quarantine; PPE donning showed a non-significant increase
31	Increase familiarity and confidence, reduce anxiety in practicing universal precautions for NSI^r^ prevention, and reduce NSI incidence during the internship	VR game-based training for occupational NSI prevention	Unity 3D	Immersive VR scenarios, random scenarios with safe/unsafe behaviors, real-time feedback, performance assessment, and elements of surprise and uncertainty	The VR training improved accuracy, decreased anxiety, and enhanced familiarity and confidence in NSI prevention, with better retention observed two months post training
32	Enhancing standard precautions	N/A	Mobile phones	Interactive game with scenarios, video demos, made in Adobe Flash	Online education and VR games significantly improved nursing students’ performance
33	Evaluating attitudes, knowledge, and adoption of the influenza vaccine	Flu Bee Game	HTML5 web application, accessible via web browsers on any device	Interactive influenza questions, build ’honeycomb path,' myth-busting, feedback, leaderboards	The game can enhance understanding and increase the likelihood of vaccination
34	Enhancing empathy, emphasizing immersion, and perspective	That Dragon, Cancer - a narrative-focused video game	VR (Oculus Go) and non-VR (Dell laptops, iPads)	VR versus non-VR, role perspectives, interactive emotional scenarios	The results of this study demonstrate that role-playing games are viable for nursing education, with insights into empathy training
35	raising awareness about stigmatizing attitudes toward individuals with serious mental disorders	N/A	Online, using the Genial.ly digital platform	Teams of 4 solve linear puzzles in a 60 min escape room, exploring the life of a person with serious mental illness, reflecting real-life challenges like stigma and medication side effects	Virtual escape rooms can serve as an effective method for educating health sciences students
36	To improve students’ attitudes towards learning and enhance their game flow experience related to safe medication use in older adults	N/A	Physical venue designed as a geriatric nursing training room	Immersive escape room experience with puzzles, time constraints, and a narrative involving medication safety for older adults	The ER game significantly improved learning attitudes and game flow experience in the test group compared to the control group
37	learning of nursing care in pediatrics	Serious game for pediatric nursing practice	VTS Editor Education version 2 (game design), VTS Player (access), VTS Perform (progress tracking)	Three scenarios (gastric feeding, peripheral venous access, bronchial aspiration), scoring system, feedback, performance tracking, attempts, and error recording	The game significantly improved clinical knowledge and motivation, with students showing high satisfaction and engagement
38	Enhancing delegation skills	Delegation Poker	Classroom-based, using physical cards and a board	Students used modified poker cards to select delegation strategies in nursing scenarios, earning competitive points	The game was an engaging and educational tool that successfully taught nursing students the nuances of delegation in a fun and interactive manner

a Not applicable.

b GBL: game-based learning.

c NCLEX: National Council Licensure Examination.

d SBAR: Situation, Background, Assessment, and Recommendation.

e CR: clinical reasoning.

f SG: serious game.

g TT: traditional teaching.

h CPR: cardiopulmonary resuscitation.

i VR: virtual reality.

j NTS: nontechnical skills.

k IVR: immersive virtual reality.

l PU-VRSim: pressure ulcer management virtual reality simulation.

m PU: pressure ulcer.

n BAM: burn assessment mission.

o ECG: electrocardiography.

p RPG: role-playing game.

q PPE: personal protective equipment.

r NSI: needle stick or sharp injuries.

### Quality Assessment and Bias Risk Evaluation

To enhance the quality, 3 authors independently evaluated the eligible studies for risk of bias to minimize any probable bias risk using the NOS risk assessment tool (Table 3). Worthy to mention that a total score of 9 in 3 categories is calculated in this numerical bias assessment tool. These three categories include selection, comparability, and exposure or outcome. Numerical values of 4, 2, and 3 are attributed to these categories, respectively. A maximum scale of 9 is going to be expected for each of the included studies by summing these numbers together.

**Table 3.** Newcastle-Ottawa Scale bias risk assessment of the study. Good quality: 3 or 4 stars in selection domain AND 1 or 2 stars in comparability domain AND 2 or 3 stars in exposure or outcome domain; fair quality: 2 stars in selection domain AND 1 or 2 stars in comparability domain AND 2 or 3 stars in exposure or outcome domain; poor quality: 0 or 1 star in selection domain OR 0 stars in comparability domain OR 0 or 1 stars in exposure or outcome domain.

ID	Selection (out of 4)	Comparability (out of 2)	Exposure/Outcome (out of 3)	Total (Out of 9)
1	3	2	3	8
2	4	2	3	9
3	2	1	3	6
4	4	2	3	9
5	3	2	2	7
6	3	2	2	7
7	3	2	3	8
8	2	2	3	7
9	3	2	3	8
10	4	1	2	7
11	3	2	3	8
12	4	2	3	9
13	3	2	3	8
14	3	1	2	6
15	4	2	3	9
16	3	2	3	8
17	3	2	3	8
18	2	1	2	5
19	3	2	2	7
20	3	2	2	7
21	3	2	3	8
22	4	2	3	9
23	3	2	3	8
24	4	2	3	9
25	3	0	3	6
26	4	2	2	8
27	1	2	3	6
28	2	1	3	6
29	4	2	3	9
30	3	0	3	6
31	3	2	3	8
32	3	1	3	7
33	4	2	3	9
34	4	2	3	9
35	2	1	2	5
36	3	1	3	7
37	2	2	2	6
38	2	1	3	6

### Results

### Data Sources

In this study, 801 articles were initially retrieved using the specified search strategy in the databases of PubMed, Embase, Scopus, and Web of Science.

### Study Selection

**Figure 1.**
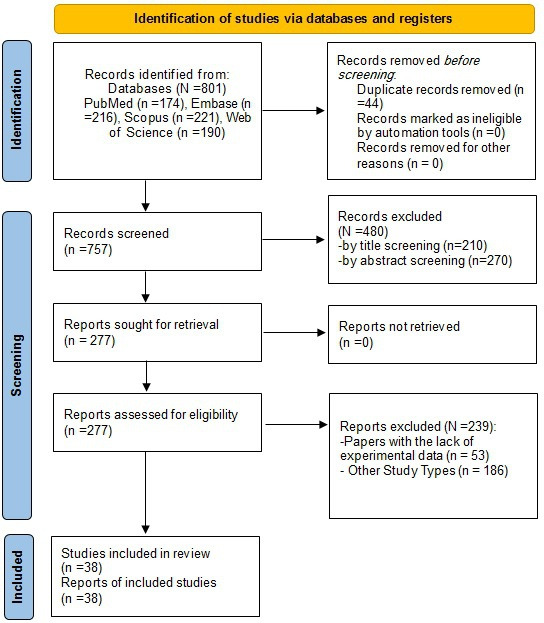
PRISMA (Preferred Reporting Items for Systematic Reviews and Meta-Analyses) 2020 flow diagram of the study retrieval process.

In total, 44 duplicate articles were removed, and 757 studies were screened. After applying the inclusion and exclusion criteria, 719 articles were excluded, leaving 38 articles for full-text review (Figure 1).

### Data Extraction

**Figure 2.**
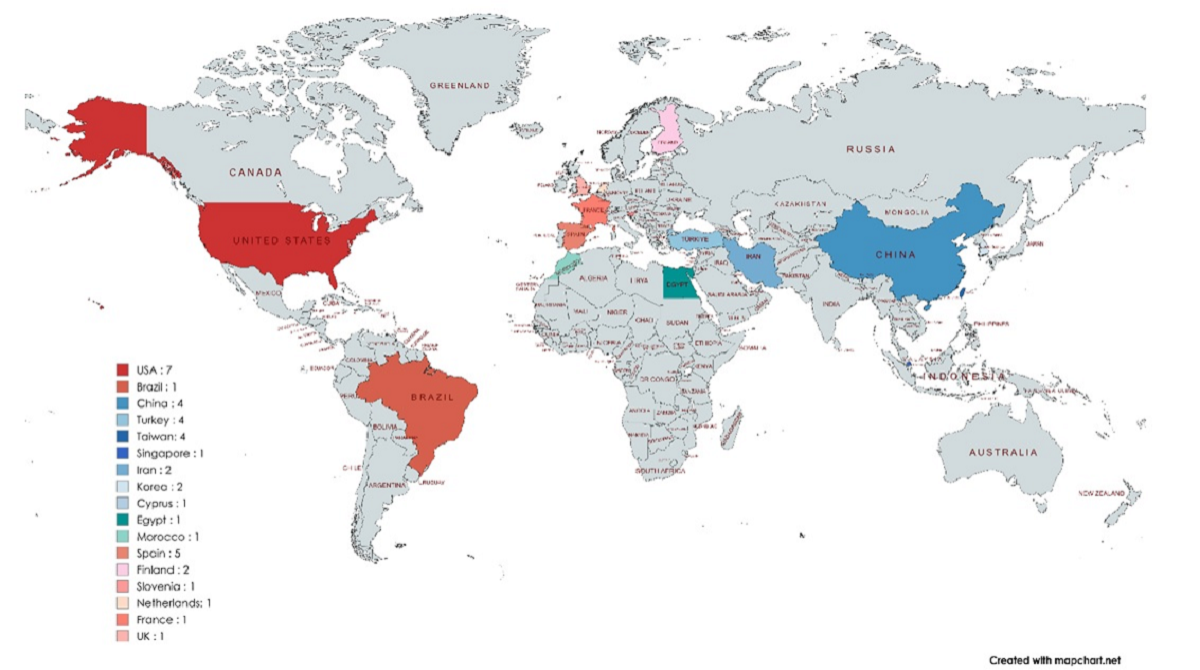
Geographical distribution of the countries where the studies were conducted.

**Figure 3.**
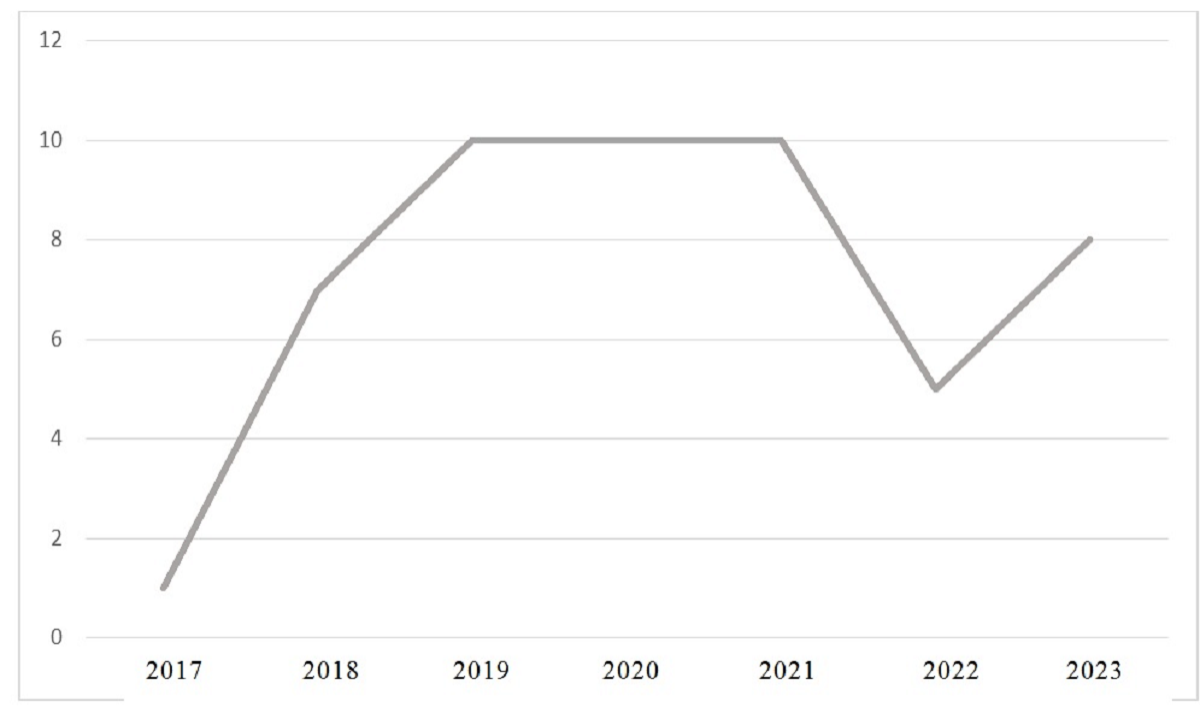
Overview of study conduction years.

The included articles were conducted in the countries of Brazil (n=1), Korea (n=2), Turkey (n=4), China (n=4), Netherlands (n=1), United States (n=7), Taiwan (n=3), Egypt (n=1), Iran (n=2), Spain (n=5), Morocco (n=1), Finland (n=1), Singapore (n=1), United Kingdom (n=1), France (n=1), Cyprus (n=1), and Slovenia (n=1) and were published between 2018 and 2023 (Figure 2). The final included studies were conducted over a range of years, from 2017 to 2023. Figure 3 provides a visual summary of these timeframes.

### Quality Assessment and Bias Risk Evaluation

As shown in Table 3, according to the NOS risk assessment tool, out of 38 included articles, 36 articles were of good quality (≥6), 2 articles were of fair quality (5≥, >2), and none of the articles were of poor quality (2≥).

### Main Findings

**Figure 4.**
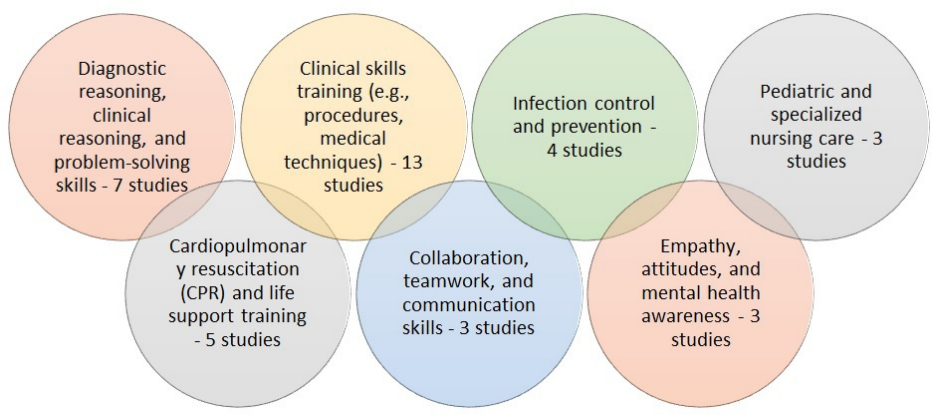
Overview of studies’ objectives.

Our results showed that several learning objectives can be conducted by GBL. These objectives included developing diagnostic reasoning, enhancing knowledge and cognitive skills, and improving training methods (Figure 4).

**Figure 5.**
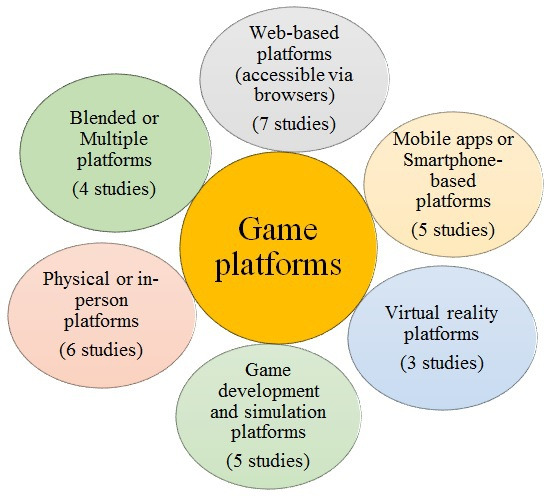
Overview of game platforms.

**Figure 6.**
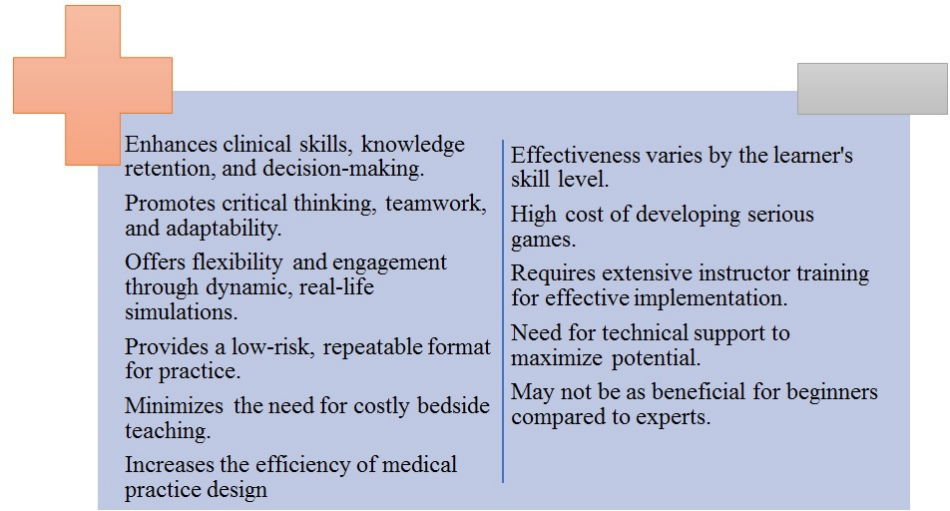
Summary of the benefits and limitations of game-based learning in nursing education.

**Figure 7.**
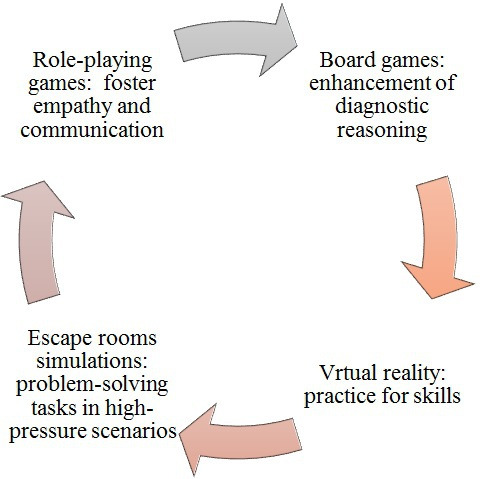
Platforms and game types associated with specific learning objectives in nursing education. VR: virtual reality.

We also showed that different platforms were used for the games. While many games used web-based platforms, few were conducted in person, and some were developed in-app formats for smartphones. Game platforms are compiled in Figure 5, providing an overview of the different game platforms. In addition, while we summarized the 3 key terms of GBL and its benefits and limitations in Figure 6, we illustrated how various platforms and game types aligned with distinct learning objectives in nursing education in Figure 7.

Finally, multiple studies were conducted in the Asia-Pacific region, where we provided a more in-depth analysis of the region’s unique approaches to nursing education and the integration of technology. These studies, involving Asia-Pacific Islander Nursing (APIN) student populations from countries such as Korea, China, and Singapore, highlighted strong engagement with mobile and virtual reality (VR)–based GBL tools. These studies also reported improvements in clinical skills, knowledge retention, and student motivation following the use of these technologies.

## Discussion

### Principal Findings

We showed that a broad range of learning objectives, such as developing diagnostic reasoning, enhancing knowledge and cognitive skills, and improving training methods, can be conducted by a game-based platform. We also showed that while many games used web-based platforms, few were conducted in person, and some were developed in app formats for smartphones. The emergence of game-based learning in recent years has led to significant changes in the health education system and curricula [52]. These methods are gaining considerable attention for their potential to boost student motivation and engagement in the learning process. The educators are offered new novel methods to enhance the education process, specifically medical education, by the premise of using serious games and gamification of the learning processes.

### Overview of Studies and Learning Objectives

After a thorough evaluation of 38 studies, our results indicated that the most frequent year for conducting studies was 2023, with contributions from countries like Brazil, Korea, and the United States. The United States contributed the most studies, covering topics such as sterile catheter insertion, diagnostic reasoning, and empathy training. The main learning included developing diagnostic reasoning, enhancing nursing knowledge and cognitive skills, and improving practical training methods such as cardiopulmonary resuscitation (CPR) and tracheostomy care. In addition, a focus on fostering student engagement, motivation, critical thinking, and collaborative problem-solving. Various platforms were used, ranging from web-based applications like Moodle and Amazon Web Services to advanced VR tools such as HTC Vive Cosmos and mobile apps. The key findings demonstrated that serious games and VR simulations were highly effective in boosting nursing students’ knowledge, practical skills, and confidence. These techniques also enhanced collaborative problem-solving, critical thinking, and motivation, with significant improvements in clinical reasoning and knowledge retention.

### GBL and the Range of Targeted and Enhanced Skills

Studies from Brazil and Korea demonstrate that GBL effectively enhances diagnostic reasoning and CPR skills through more engaging and interactive approaches than traditional methods [19,21]. These findings are reinforced by research across countries, including Turkey, where games improved tracheostomy care, and the Netherlands, which highlighted the development of collaborative problem-solving skills through serious games [26,39]. For developing skills for high-risk medical procedures, such as extracorporeal membrane oxygenation pipeline handling and pressure ulcer management, the eligible studies used serious games [30,37]. VR-based learning, as studied in the United States and China, enables students to practice sterile techniques and clinical care in immersive environments, boosting their confidence and competence [27,38]. In addition, studies have shown how serious games can rapidly adapt to global challenges, such as improving COVID-19 protocols [42]. Finally, escape-room games have proven effective in fostering clinical reasoning, collaboration, and reducing stigma toward mental health disorders [47].

Overall, the range of skills enhanced through educational games in nursing includes technical procedures, cognitive decision-making, communication, teamwork, and professional empathy, demonstrating the broad and versatile impact of GBL. Although some of these studies were conducted in western contexts, the core educational benefits are equally relevant and transferable to Asia-Pacific nursing students.

### Platforms’ and Game Types’ Association With Learning Objectives

According to our results, the following platforms and game types are linked to distinct learning objectives in nursing education:

1. Diagnostic reasoning skills games: Board games like “Enfermeiro Diagnosticador” and web-based tools enhance diagnostic reasoning [13].
2. CPR and life support training: Web-based and mobile games like mobile CPR improve CPR training through interactive simulations [13,15,23]. In addition, mobile apps like Guess It and gamified flipped classrooms enhance knowledge retention in life support and nursing fundamentals with features such as quizzes and leaderboards [24,25].
3. Clinical procedural skills: Virtual reality platforms, such as virtual reality sterile urinary catheter insertion game and pressure ulcer management virtual reality simulation, offer immersive practice for skills like catheter insertion and pressure ulcer management [27,30].
4. Teamwork, clinical competency, and emergency skills: Escape rooms and virtual emergency room simulations focus on teamwork and clinical competency by presenting problem-solving tasks in high-pressure scenarios [20,40,48].
5. Specialized skills: Role-playing games improve specialized skills, such as electrocardiogram interpretation, and foster empathy and communication [36,38].

These diverse platforms effectively align with educational goals, combining interactive, scenario-based learning with feedback and collaboration to enhance student engagement, clinical skills, and decision-making. Figure 7 illustrates how various platforms and game types align with distinct learning objectives in nursing education.

Depending on the game formats, these games show strong potential for adaptation across Asia-Pacific contexts. While VR and web-based platforms suit technologically advanced regions and might require more complex infrastructure, mobile games and gamified classrooms may be more feasible for underresourced regions of APIN settings.

### Impact of GBL on Learning Outcomes and Their Broader Discussion in Nursing Education

The comparative value of simulation-based gaming versus traditional teaching methods in nursing education demonstrates the significant benefits of serious games in enhancing clinical skills, knowledge retention, and decision-making, which aligns with the previously mentioned facts from the available literature in this sector. These studies demonstrate that simulation-based gaming provides a dynamic, effective alternative to traditional methods, offering nursing students not only technical proficiency but also the critical thinking, teamwork, and decision-making skills needed in modern health care environments. These games offer flexibility, engagement, and the ability to simulate real-life scenarios in a low-risk, repeatable format, making them a valuable tool in nursing education. Ultimately, this novel method has significant potential to minimize costly bedside teaching and enhance the efficiency of medical practice design [1]. Ultimately, GBL represents a novel and transformative method that has the potential to enhance the efficiency and effectiveness of nursing education globally. Furthermore, for APIN students, GBL can offer an effective way to bridge gaps caused by limited clinical placements or cultural and language barriers. Their adaptability makes them especially valuable in diverse Asia-Pacific settings.

### Challenges

Although GBL methods emerge as effective and beneficial methods, there are still significant challenges and limitations to their use [1]. One major issue is the “expertise reversal effect,” which highlights how the effectiveness of serious games varies based on the learner’s skill level. For instance, while these games may be beneficial for experts, they may not be as useful for beginners. Ensuring that new learners can still benefit from the game is crucial to their success [53]. Developing adaptive games that can adjust difficulty level according to player level can emerge as an effective solution to this challenge. Another challenge is the high cost of developing serious games, which can run into thousands of dollars. Creating these educational tools requires careful consideration of both cost and efficiency to ensure they remain feasible for widespread use [1]. Creation of games in collaboration with institutes and seeking sponsorship or grants can reduce the development costs. In addition to financial constraints, the need for training instructors to effectively implement the games and providing adequate technical support are common hurdles. Without proper training and support, the potential of serious games may not be fully realized [54]. Establishment of technical support teams and training standardized instructors can maximize the benefits of these games.

One major issue is the “expertise reversal effect,” which highlights how the effectiveness of serious games varies based on the learner’s skill level. For instance, while these games may be beneficial for experts, they may not be as useful for beginners. Ensuring that new learners can still benefit from the game is crucial to their success [53]. Developing adaptive games that can adjust difficulty level according to player level can emerge as an effective solution to this challenge.

In addition to financial constraints, the need for training instructors to effectively implement the games and providing adequate technical support are common hurdles. Without proper training and support, the potential of serious games may not be fully realized [54]. Establishment of technical support teams and training standardized instructors can maximize the benefits of these games.

Limited funding and access to professional developers, along with a lack of trained educators, can complicate these issues further and lead to a much more challenging situation in underresourced Asia-Pacific regions. Addressing these challenges is essential to making serious games a more practical and accessible tool in educational settings, particularly in medical training, where their application has the potential to significantly enhance learning outcomes if used correctly. Figure 6 provides a summary of the discussed benefits and limitations. Finally, it is noteworthy to mention that game-based learning in nursing is a novel and beneficial method, and by addressing its limitations, we hope it becomes more widespread in the future. Finally, it is noteworthy to mention that game-based learning in nursing is a novel and beneficial method, and by addressing its limitations, we hope it becomes more widespread in the future.

Lastly, it is noteworthy to mention that game-based learning in nursing is a novel and beneficial method, and by addressing its limitations, we hope it becomes more widespread in the future.

### Asia-Pacific Approaches to Technology-Enhanced Nursing Education

One group of the included studies was conducted in Asia-Pacific countries, which mainly originated from Korea, Taiwan, China, and Singapore, alongside Iran. These studies highlight a regional trend of incorporating advanced digital technologies in nursing education. A wide range of platforms was used in this section of the literature, including VR technologies, web-based platforms, mobile apps, and virtual environments. The primary goals of these studies encompassed a wide range of areas, including clinical skills development, critical thinking enhancement, infection control practices, CPR training, and emergency care [21,22,29,30,33,35,37,40,43,48,55]. According to these findings, in this region, there is a strong emphasis on innovation, rapid adoption of new technologies, and a cultural value placed on education and professional development. Understanding these dynamics highlights the region’s distinct approach to advancing nursing education through technology-driven methods. However, the development, adoption, and effectiveness of GBL can be influenced by cultural factors prevalent in the Asia-Pacific context. One issue is that in collectivist societies, common in many parts of Asia, students may prioritize group harmony and respect for authorities, which can affect their engagement in interactive and competitive learning environments like GBL [56,57]. Since Western GBL approaches often emphasize individualism and assertiveness, they may not fully align with the values common in Asia-Pacific contexts. Adapting game content to focus more on collaboration, respect for authority, and cultural relevance can improve acceptance among APIN students. While this situation provides a challenging base for the implementation of GBL, the acceptance of these learning methods also varies across different cultures of Asia-Pacific countries, necessitating culturally sensitive designs and strategies. Ultimately, in low-resource regions of this area, limited access to technological infrastructure can pose challenges to the widespread adoption of GBL [58].

### Limitations and Future Prospects

This study has several limitations. First, it only included English-language publications, potentially omitting valuable insights from non-English studies, which may affect the comprehensiveness of our findings. Including multilingual studies can highlight local innovations in many aspects, such as region-specific game designs or unique instructional methods. In addition, publication bias could skew results since positive studies are more likely to be published. Variability in definitions and classifications of GBL and a lack of standardized measurement tools complicate data interpretation. Finally, our research did not explore the long-term effects of GBL on nursing competencies and patient outcomes, which necessitates longitudinal studies for a comprehensive understanding. Looking ahead, future research should aim to include multilingual studies to broaden perspectives on GBL in nursing education. Longitudinal studies are essential to assess the enduring impacts of GBL on clinical competencies and patient care outcomes. In addition, developing standardized assessment tools will help unify definitions and improve the comparability of research findings, ultimately enhancing the evidence base for GBL’s effectiveness.

### Conclusion

To conclude, the premise of GBL and its infiltration into nursing education represents a novel yet challenging approach that enhances student engagement, motivation, and clinical skills. The feasibility and benefits of this method lead to educators creating immersive learning environments that promote critical thinking and collaborative problem-solving. However, like any other new approach, it introduces a few challenges to the education system. Addressing these limitations is crucial for maximizing the potential of GBL in nursing education. As the field continues to evolve, future research should aim to broaden perspectives through multilingual studies and assess the long-term impacts of GBL on nursing competencies and patient care outcomes. Overall, the promising benefits of GBL could significantly enhance the quality of nursing education and ultimately improve health care delivery. In the APIN, implementing GBL requires sensitivity to cultural values. Educators should design collaborative, culturally aligned approaches and strategies, plus ensure accessibility despite resource limitations.

## Supplementary material

10.2196/70737 Multimedia Appendix 1 Search query of databases.

10.2196/70737 Checklist 1 PRISMA (Preferred Reporting Items for Systematic Reviews and Meta-Analyses) 2020 checklist.

## Abbreviation

APIN: Asia-Pacific Islander Nursing
GBL: game-based learning
NOS: Newcastle-Ottawa Scale
PRISMA: Preferred Reporting Items for Systematic Reviews and Meta-Analyses
VR: virtual reality
VRSUCIG: virtual reality sterile urinary catheter insertion game

## References

[1] Xu Y, Lau Y, Cheng LJ, Lau ST (2021). Learning experiences of game-based educational intervention in nursing students: a systematic mixed-studies review. Nurse Educ Today.

[2] Esmail S, Concannon B (2025). Immersive virtual reality and AI (generative pretrained transformer) to enhance student preparedness for objective structured clinical examinations: mixed methods study. JMIR Serious Games.

[3] Martín-Valero R, Vega-Morales A, Martín-Vega FJ, Rodriguez-Huguet M, Rodríguez-Martínez MC, Vinolo-Gil MJ (2025). Effectiveness of augmented reality in the teaching of health university students: quasi-experimental study. JMIR Serious Games.

[4] Kuruca Ozdemir E, Dinc L (2022). Game-based learning in undergraduate nursing education: a systematic review of mixed-method studies. Nurse Educ Pract.

[5] Lee M, Shin S, Lee M, Hong E (2024). Educational outcomes of digital serious games in nursing education: a systematic review and meta-analysis of randomized controlled trials. BMC Med Educ.

[6] Safeena Beevi SS, Veragi OP (2023). Game based learning in nursing - an innovative strategy. Medicon Med Sci.

[7] Tavares N (2022). The use and impact of game-based learning on the learning experience and knowledge retention of nursing undergraduate students: a systematic literature review. Nurse Educ Today.

[8] Lopreiato JO (2025). Healthcare simulation dictionary.

[9] Pan L, Tlili A, Li J (2021). How to implement game-based learning in a smart classroom? A model based on a systematic literature review and delphi method. Front Psychol.

[10] Xu M, Luo Y, Zhang Y, Xia R, Qian H, Zou X (2023). Game-based learning in medical education. Front Public Health.

[11] Gudadappanavar AM, Benni JM, Javali SB (2021). Effectiveness of the game-based learning over traditional teaching-learning strategy to instruct pharmacology for Phase II medical students. J Educ Health Promot.

[12] Wan K, King V, Chan K (2021). Examining essential flow antecedents to promote students’ self-regulated learning and acceptance of use in a game-based learning classroom. EJEL.

[13] Tinôco J, Silva LSD, de Medeiros TM (2023). Enfermeiro Diagnosticador” board game for teaching diagnostic reasoning in nursing: a quasi-experimental study. Acta Paulista de Enfermagem.

[14] Vázquez-Calatayud M, García-García R, Regaira-Martínez E, Gómez-Urquiza J (2024). Real-world and game-based learning to enhance decision-making. Nurse Educ Today.

[15] Morgan DJ, Scherer L, Pineles L (2024). Game-based learning to improve diagnostic accuracy: a pilot randomized-controlled trial. Diagnosis (Berl).

[16] Morrell B, Eukel HN (2021). Shocking escape: a cardiac escape room for undergraduate nursing students. Simul Gaming.

[17] Morrell BLM, Eukel HN, Santurri LE (2020). Soft skills and implications for future professional practice: qualitative findings of a nursing education escape room. Nurse Educ Today.

[18] Kubin L (2020). Using an escape activity in the classroom to enhance nursing student learning. Clin Simul Nurs.

[19] Blanié A, Amorim MA, Benhamou D (2020). Comparative value of a simulation by gaming and a traditional teaching method to improve clinical reasoning skills necessary to detect patient deterioration: a randomized study in nursing students. BMC Med Educ.

[20] Gómez-Urquiza JL, Hueso-Montoro C, Correa-Rodríguez M (2022). Nursing students’ experience using an escape room for training clinical skills and competencies on emergency care: A qualitative observational study. Medicine (Baltimore).

[21] Baek G, Lee E (2024). Development and effects of advanced cardiac resuscitation nursing education program using web-based serious game: application of the IPO model. BMC Nurs.

[22] Farsi Z, Yazdani M, Butler S, Nezamzadeh M, Mirlashari J (2021). Comparative effectiveness of simulation versus serious game for training nursing students in cardiopulmonary resuscitation: a randomized control trial. International Journal of Computer Games Technology.

[23] Fijačko N, Masterson Creber R, Metličar Š (2024). Effects of a serious smartphone game on nursing students’ theoretical knowledge and practical skills in adult basic life support: randomized wait list-controlled trial. JMIR Serious Games.

[24] Gutiérrez-Puertas L, García-Viola A, Márquez-Hernández VV, Garrido-Molina JM, Granados-Gámez G, Aguilera-Manrique G (2021). Guess it (SVUAL): an app designed to help nursing students acquire and retain knowledge about basic and advanced life support techniques. Nurse Educ Pract.

[25] Elzeky MEH, Elhabashy HMM, Ali WGM, Allam SME (2022). Effect of gamified flipped classroom on improving nursing students’ skills competency and learning motivation: a randomized controlled trial. BMC Nurs.

[26] Biyik Bayram Ş, Çalişkan N, Gülnar E (2023). The effect of web-based tracheostomy care game on nursing students’ knowledge levels and their views of the process. Clinical and Experimental Health Sciences.

[27] Breitkreuz KR, Kardong-Edgren S, Gilbert GE (2021). A multi-site study examining the usability of a virtual reality game designed to improve retention of sterile catheterization skills in nursing students. Simul Gaming.

[28] Chan K, Kor PPK, Liu JYW, Cheung K, Lai T, Kwan RYC (2024). The use of immersive virtual reality training for developing nontechnical skills among nursing students: multimethods study. Asian Pac Isl Nurs J.

[29] Hwang GJ, Chang CY (2020). Facilitating decision-making performances in nursing treatments: a contextual digital game-based flipped learning approach. Interact Learn Environ.

[30] Jung SY, Moon KJ (2024). Pressure ulcer management virtual reality simulation (PU-VRSim) for novice nurses: mixed methods study. JMIR Serious Games.

[31] Koivisto JM, Buure T, Engblom J, Rosqvist K, Haavisto E (2024). Association between game metrics in a simulation game and nursing students’ surgical nursing knowledge - a quasi-experimental study. BMC Nurs.

[32] Koivisto JM, Buure T, Engblom J, Rosqvist K, Haavisto E (2024). The effectiveness of simulation game on nursing students’ surgical nursing knowledge—a quasi-experimental study. Teach Learn Nurs.

[33] Lau ST, Liaw SY, Loh WL (2023). Mid-career switch nursing students’ perceptions and experiences of using immersive virtual reality for clinical skills learning: a mixed methods study. Nurse Educ Today.

[34] Kulakaç N, Çilingir D (2024). The effect of a serious game-based web application on stoma care education for nursing students: a randomized controlled trial. Teaching and Learning in Nursing.

[35] Nasirzade A, Deldar K, Froutan R, Shakeri MT (2024). Comparison of the effects of burn assessment mission game with feedback lecture on nursing students’ knowledge and skills in the burn patients’ assessment: a randomized clinical trial. BMC Med Inform Decis Mak.

[36] Karcı HD, Bektaş Akpınar N, Özcan Yüce U (2024). Role-play based gamification for communication skills and nursing competence in internal medicine nursing. Journal of Nursology.

[37] Wang Z, Gu R, Wang J (2022). Effectiveness of a game-based mobile application in educating intensive critical care specialist nurses on ECMO pipeline preflushing: a quasi-experimental trial (preprint). JMIR Serious Games.

[38] Chang CY, Kao CH, Hwang GJ, Lin FH (2019). From experiencing to critical thinking: a contextual game-based learning approach to improving nursing students’ performance in Electrocardiogram training. Educ Technol Res Dev.

[39] de Beer E, van Os-Medendorp HH, Groeneveld S, Jukema J (2023). Perceived contribution of a hybrid serious game to the development of collaborative problem solving among undergraduate nursing students: a mixed method design. Nurse Educ Pract.

[40] Wong JYH, Ko J, Nam S (2022). Virtual ER, a serious game for interprofessional education to enhance teamwork in medical and nursing undergraduates: development and evaluation study. JMIR Serious Games.

[41] Ropero-Padilla C, Rodriguez-Arrastia M, Martinez-Ortigosa A, Salas-Medina P, Folch Ayora A, Roman P (2021). A gameful blended-learning experience in nursing: a qualitative focus group study. Nurse Educ Today.

[42] Calik A, Cakmak B, Kapucu S, Inkaya B (2022). The effectiveness of serious games designed for infection prevention and promotion of safe behaviors of senior nursing students during the COVID-19 pandemic. Am J Infect Control.

[43] Wu SH, Huang CC, Huang SS (2020). Effect of virtual reality training to decreases rates of needle stick/sharp injuries in new-coming medical and nursing interns in Taiwan. J Educ Eval Health Prof.

[44] Al-Mugheed K, Bayraktar N, Al-Bsheish M (2022). Effectiveness of game-based virtual reality phone application and online education on knowledge, attitude and compliance of standard precautions among nursing students. PLoS One.

[45] Mitchell G, Leonard L, Carter G, Santin O, Brown Wilson C (2021). Evaluation of a “serious game” on nursing student knowledge and uptake of influenza vaccination. PLoS One.

[46] Ma Z, Huang KT, Yao L (2021). Feasibility of a computer role-playing game to promote empathy in nursing students: the role of immersiveness and perspective. Cyberpsychol Behav Soc Netw.

[47] Rodríguez-Ferrer JM, Manzano-León A, Cangas AJ (2022). Acquisition of learning and empathy towards patients in nursing students through online escape room: an exploratory qualitative study. Psychol Res Behav Manag.

[48] Chen D, Liu F, Zhu C, Tai C, Zhang Y, Wang X (2023). The effect of an escape room game on college nursing students’ learning attitude and game flow experiences in teaching safe medication care for the elderly: an intervention educational study. BMC Med Educ.

[49] EL Machtani EL Idrissi W, Chemsi G, EL Kababi K, Radid M (2022). The impact of serious game on the nursing students’ learning, behavioral engagement, and motivation. Int J Emerg Technol Learn.

[50] Labrague L (2024). Delegation poker game in nursing. J Nurs Educ.

[51] Trakeostomi Bakımı.

[52] Mehraeen E, Dashti M, Mirzapour P (2025). Serious games in nursing education: a systematic review of current evidence. International Journal of Africa Nursing Sciences.

[53] Dankbaar M (2017). Serious games and blended learning; effects on performance and motivation in medical education. Perspect Med Educ.

[54] Oestreich JH, Guy JW (2022). Game-based learning in pharmacy education. Pharmacy (Basel).

[55] Chang CY, Chung MH, Yang JC (2022). Facilitating nursing students’ skill training in distance education via online game-based learning with the watch-summarize-question approach during the COVID-19 pandemic: a quasi-experimental study. Nurse Educ Today.

[56] Jossan KS, Gauthier A, Jenkinson J (2021). Cultural implications in the acceptability of game-based learning. Comput Educ.

[57] McNiesh SG (2015). Cultural norms of clinical simulation in undergraduate nursing education. Glob Qual Nurs Res.

[58] Kelly MA, Balakrishnan A, Naren K (2018). Cultural considerations in simulation-based education. Asia Pac Scholar.

