# Supplementary material for: Games to Improve the Clinical Skills of Nursing Students: Systematic Review of Current Evidence
Source: Asian Pac Isl Nurs J. 2025 Aug 11;9:e70737. doi: 10.2196/70737 (PMC12338851; doi:10.2196/70737)
Supplement: Multimedia Appendix 1 [file apinj-v9-e70737-s001.pdf]

Educational game to improve the clinical skills of nursing students

Pubmed Search Query

Time of search: 21-Jul-2024

Results: 174

(("games, experimental"[MeSH Terms] OR "gamification"[MeSH Terms] OR "game"[Title/Abstract] OR "gamification"[Title/Abstract] OR "educational game"[Title/Abstract] OR "serious game"[Title/Abstract]) AND ("Nurses"[MeSH Terms] OR "Nursing"[MeSH Terms] OR "Nurse Practitioners"[MeSH Terms] OR Nurses[Ti] OR Nursing[Ti] OR "Nurse Practitioners"[Title/Abstract]) AND ("Education"[MeSH Terms] OR "Education"[Title/Abstract] OR "Learning"[Title/Abstract] OR "Nursing education"[Title/Abstract] OR "Clinical skills"[Title/Abstract] OR "Virtual reality"[Title/Abstract] OR "Virtual learning"[Title/Abstract] OR "Virtual education"[Title/Abstract] OR "Virtualization"[Title/Abstract]))

Embase Search Query

Time of search: 21-Jul-2024
Results: 216

(('game'/exp OR 'gamification'/exp OR 'game':ti,ab,kw OR 'gamification':ti,ab,kw OR 'educational game':ti,ab,kw OR 'serious game':ti,ab,kw) AND ('nurse'/exp OR 'nursing'/exp OR 'nurse practitioner'/exp OR 'nurses':ti OR 'nursing':ti OR 'nurse practitioners':ti,ab,kw) AND ('education'/exp OR 'education':ti,ab,kw OR 'learning':ti,ab,kw OR 'nursing education':ti,ab,kw OR 'clinical skills':ti,ab,kw OR 'virtual reality':ti,ab,kw OR 'virtual learning':ti,ab,kw OR 'virtual education':ti,ab,kw OR 'virtualization':ti,ab,kw))

Scopus Search Query

Time of search: 21-Jul-2024
Results: 221

(TITLE-ABS ("experimental games" OR "gamification" OR "game" OR "educational game" OR "serious game") AND TITLE ("nurses" OR "nursing" OR "nurse practitioners") AND TITLE-ABS ("education" OR "learning" OR "nursing education" OR "clinical skills" OR "virtual reality" OR "virtual learning" OR "virtual education" OR "virtualization"))

Web of science Search Query

Time of search: 21-Jul-2024
Results: 190

(TS=("experimental games" OR "gamification" OR "game" OR "educational game" OR "serious game") AND TS=("nurses" OR "nursing" OR "nurse practitioners") AND TS=("education" OR "learning" OR "nursing education" OR "clinical skills" OR "virtual reality" OR "virtual learning" OR "virtual education" OR "virtualization"))
